# Supplementary material for: A Critical View of the Application of the APEX Software (Aqueous Photochemistry of Environmentally-Occurring Xenobiotics) to Predict Photoreaction Kinetics in Surface Freshwaters
Source: Molecules. 2019 Dec 18;25(1):9. doi: 10.3390/molecules25010009 (PMC7017383; doi:10.3390/molecules25010009)
Supplement: Supplementary file 1 [file molecules-25-00009-s001.zip › 3.2.4_gcc-4.4.0.zip!/3.2.4_gcc-4.4.0/3.2.4_gcc-4.4.0/doc/octave/html/faq/Octave-FAQ.html]

Frequently asked questions about Octave (with answers)


# Frequently asked questions about Octave (with answers)

---

Next: What is Octave?,
Up: (dir)

## Frequently asked questions about Octave (with answers)

## Preface

This is a list of frequently asked questions (FAQ) for Octave users.

We are always looking for new questions (*with* answers), better
answers, or both. Please send suggestions to bug@octave.org.
If you have general questions about Octave, or need help for something
that is not covered by the Octave manual or the FAQ, please use the
help@octave.org mailing list.

This FAQ is intended to supplement, not replace, the Octave manual.
Before posting a question to the help@octave.org mailing list,
you should first check to see if the topic is covered in the manual.

- What is Octave?- Licensing Issues- How can I cite Octave?- Series 3.0.N- Octave Features- Learning more about Octave- Getting Octave- Installation- Common problems- How do I ...?- MATLAB compatibility- Index

---

Next: Licensing Issues,
Previous: Top,
Up: Top

## 1 What is Octave?

Octave is a high-level interactive language, primarily intended for
numerical computations that is mostly compatible with
Matlab.^1^

Octave can do arithmetic for real, complex or integer-valued scalars
and matrices, solve sets of nonlinear algebraic equations, integrate
functions over finite and infinite intervals, and integrate systems of
ordinary differential and differential-algebraic equations.

Octave uses the GNU readline library to handle reading and editing
input. By default, the line editing commands are similar to the
cursor movement commands used by GNU Emacs, and a vi-style line
editing interface is also available. At the end of each session, the
command history is saved, so that commands entered during previous
sessions are not lost.

The Octave distribution includes a 590+ page Texinfo manual. Access
to the complete text of the manual is available via the help command
at the Octave prompt.

- Who develops Octave?- Why GNU Octave?- What version should I use?- On what platforms does Octave run?

---

Next: Why GNU Octave?,
Up: What is Octave?

### 1.1 Who develops Octave?

Discussions about writing the software that would eventually become
Octave started in about 1988 with James B. Rawlings and John W. Eaton at
the University of Texas. John W. Eaton was the original author of
Octave, starting full-time development in February 1992. He is still
the primary maintainer. The community
of users/developers has in addition contributed some code and fuels the
discussion on the mailing lists help@octave.org (user forum),
bug@octave.org (bug reports), maintainers@octave.org
(development issues), and octave-dev@lists.sourceforge.net (all
things related to the Octave Forge repository of user-contributed
functions).

---

Next: What version should I use?,
Previous: Who develops Octave?,
Up: What is Octave?

### 1.2 Why GNU Octave?

The GNU Project was launched in 1984 to develop a complete Unix-like
operating system which is free software: the GNU system.

GNU is a recursive acronym for “GNU's Not Unix”; it is pronounced
guh-noo, approximately like canoe.

The Free Software Foundation (FSF) is the principal organizational
sponsor of the GNU Project.

Octave became GNU Octave in 1997 (beginning with version 2.0.6). This
meant agreeing to consider Octave a part of the GNU Project and support
the efforts of the FSF. However, Octave is not and has never been
developed by the FSF.

For more information about the GNU project, see www.gnu.org.

---

Next: On what platforms does Octave run?,
Previous: Why GNU Octave?,
Up: What is Octave?

### 1.3 What version should I use?

In general, you will find the latest version on
http://www.octave.org/download.html. It is
recommended to use the “testing” version of octave for general use,
and the “development” version if you want the latest features.

A list of user-visible changes since the last release is available in
the file NEWS. The file ChangeLog in the source
distribution contains a more detailed record of changes made since the
last release.

---

Previous: What version should I use?,
Up: What is Octave?

### 1.4 On what platforms does Octave run?

Octave runs on various Unices—at least Linux and Solaris, Mac OS X,
Windows and anything you can compile it on. Binary distributions exist
at least for Debian, Suse, Fedora and RedHat Linuxes (Intel and AMD
CPUs, at least), for Mac Os X and Windows' 98, 2000 and XP.

Two and three dimensional plotting is fully supported using gnuplot.

The underlying numerical solvers are currently standard Fortran ones
like Lapack, Linpack, Odepack, the Blas, etc., packaged in a library
of C++ classes. If possible, the Fortran subroutines are compiled
with the system's Fortran compiler, and called directly from the C++
functions. If that's not possible, you can still compile Octave if
you have the free Fortran to C translator f2c.

Octave is also free software; you can redistribute it and/or modify it
under the terms of the GNU General Public License as published by the
Free Software Foundation.

---

Next: How can I cite Octave?,
Previous: What is Octave?,
Up: Top

## 2 Licensing Issues

- If I write code using Octave do I have to release it under the GPL?:- Since the MEX interface allows plugins to be distributed under terms that are incompatible with the GPL, does this mean that you are encouraging people to to write non-free software for Octave?:- I wrote a program that links with Octave libraries and I don't want to release it under the terms of the GPL. Will you change the license of the Octave libraries for me?:

---

Next: Licensing MEX Files,
Up: Licensing Issues

### 2.1 If I write code using Octave do I have to release it under the GPL?

The answer depends on precisely how the code is written and how it works.

Code written entirely in the scripting language of Octave
(interpreted code in .m files) may be released under the terms of
whatever license you choose.

Code written using Octave's native plug-in interface (also known
as a .oct file) necessarily links with Octave internals and is
considered a derivative work of Octave and therefore must be
released under terms that are compatible with the GPL.

Code written using Octave's implementation of the Matlab MEX
interface may be released under the terms of whatever license you
choose, provided that the following conditions are met:

1. The plugin should not use any bindings that are specific to Octave. In
   other words, the MEX file must use the MEX interface only, and not also
   call on other Octave internals. It should be possible in principle to
   use the MEX file with other programs that implement the MEX interface
   (e.g., Matlab).- The MEX file should not be distributed together with Octave in such a
     way that they effectively create a single work. For example, you should
     not distribute the MEX file and Octave together in a single package such
     that Octave automatically loads and runs the MEX file when it starts up.
     There are other possible ways that you might effectively create a single
     work; this is just one example.

A program that embeds the Octave interpreter (e.g., by calling the
"octave\_main" function), or that calls functions from Octave's
libraries (e.g., liboctinterp, liboctave, or libcruft) is
considered a derivative work of Octave and therefore must be
released under terms that are compatible with the GPL.

---

Next: Requesting License Changes,
Previous: GPL,
Up: Licensing Issues

### 2.2 Since the MEX interface allows plugins to be distributed under terms that are incompatible with the GPL, does this mean that you are encouraging people to to write non-free software for Octave?

No. The original reason for implementing the MEX interface for Octave
was to allow Octave to run free software that uses MEX files (the
particular goal was to run SundialsTB in Octave). The intent was to
liberate that software from Matlab and increase the amount of free
software available to Octave users, not to enable people to write
proprietary code for Octave. For the good of the community, we strongly
encourage users of Octave to release the code they write for Octave
under terms that are compatible with the GPL.

---

Previous: Licensing MEX Files,
Up: Licensing Issues

### 2.3 I wrote a program that links with Octave libraries and I don't want to release it under the terms of the GPL. Will you change the license of the Octave libraries for me?

No. Instead of asking us to change the licensing terms for Octave, we
recommend that you release your program under terms that are compatible
with the GPL so that the free software community can benefit from your
work the same as you have benefitted from the work of all the people who
have contributed to Octave.

---

Next: Series 3.0.N,
Previous: Licensing Issues,
Up: Top

## 3 How can I cite Octave?

Pointing to http://www.octave.org is good, because that gives
people a direct way to find out more. If citation of a URL is not
allowed by a publisher, or if you also want to point to a traditional
reference, then you can cite the Octave manual:

```
     @BOOK{eaton:2008,
       author =     "John W. Eaton and David Bateman and Søren Hauberg",
       title =      "GNU Octave Manual Version 3",
       publisher =  "Network Theory Limited",
       year =       "2008",
       isbn =       "0-9546120-6-X"
     }
```

---

Next: Octave Features,
Previous: How can I cite Octave?,
Up: Top

## 4 What's new in version series 3.0.N and 3.1.N of Octave

The 3.0.N series has enough new features to justify a major version
number change. The 3.0.N series brings

- integer types- fixed point arithmetic- sparse matrices- Linear programming code based on GLPK- 64-bit compilation support- gzipped files and stream and consequently support of matlab v7 files- better support for both msvc and mingw- a fully compatible MEX interface- many many other minor features and compatibility changes

Here are some features that have been around since 2.1.N

- NDarrays- cells

The 3.1.N series is the current development release and will become a
3.2.N release in the future. This series brings the new features

- OpenGL backend

  An experimental OpenGL graphics backend to replace the gnuplot- Object Orient Programming- Block comments- imwrite and imread

        The functions are based on the GraphicsMagick library.- Lazy transpose

          Special treatment in the parser of things like "a' \* b", where the
          transpose is never explicitly formed but a flag is rather passed to the
          underlying LAPACK code.- Single precision type- Improved array indexing
              The underlying code used for indexing of arrays has been completely
              rewritten and so the indexing of arrays is now significantly faster.

---

Next: Learning more about Octave,
Previous: Series 3.0.N,
Up: Top

## 5 What features are unique to Octave?

- Functions defined on the command-line- Comments with #- Strings delimitted by double quotes "- Line continuation by backslash- Informative block closing- Coherent syntax- Exclamation mark as not operator- Increment and decrement operators- Unwind-protect- Built-in ODE and DAE solvers

This section refers to Matlab R2008b and Octave 2.1.51.

---

Next: Comments with #,
Up: Octave Features

### 5.1 Functions defined on the command-line

Functions can be defined by entering code on the command line, a
feature not supported by the other leading brand. For example, you may
type:

```
     octave:1> function s = hello_string (to_who)
     > ## Say hello
     > if nargin<1, to_who = "World"; end
     > s = ["Hello ",\
     >      to_who];
     > endfunction
     octave:2> hello_string ("Moon")
     ans = Hello Moon
```

---

Next: Strings delimitted by double quotes ",
Previous: Functions defined on the command-line,
Up: Octave Features

### 5.2 Comments with #

The pound character, #, may be used to start comments, in addition
to %. See the previous example. The major advantage of this is
that as # is also a comment character for unix script files, any
file that starts with a string like #! /usr/bin/octave -q will be
treated as an octave script and be executed by octave.

---

Next: Line continuation by backslash,
Previous: Comments with #,
Up: Octave Features

### 5.3 Strings delimitted by double quotes "

The double quote, ", may be used to delimit strings, in addition to
the single quote '. See the previous example. Also, double-quoted
strings include backslash interpretation (like C++, C, and Perl) while
single quoted are uninterpreted (like Matlab and Perl).

---

Next: Informative block closing,
Previous: Strings delimitted by double quotes ",
Up: Octave Features

### 5.4 Line continuation by backslash

Lines can be continued with a backslash, \, in addition to three
points .... See the previous example.

---

Next: Coherent syntax,
Previous: Line continuation by backslash,
Up: Octave Features

### 5.5 Informative block closing

You may close `function`, `for`, `while`, `if`,
... blocks with `endfunction`, `endfor`, `endwhile`,
... keywords in addition to using `end`. As with Matlab, the
`end` (or `endfunction`) keyword that marks the end of a
function defined in a .m file is optional.

---

Next: Exclamation mark as not operator,
Previous: Informative block closing,
Up: Octave Features

### 5.6 Coherent syntax

Indexing other things than variables is possible, as in:

```
     octave:1> [3 1 4 1 5 9](3)
     ans = 4
     octave:2> cos([0 pi pi/4 7])(3)
     ans = 0.70711
```

---

Next: Increment and decrement operators,
Previous: Coherent syntax,
Up: Octave Features

### 5.7 Exclamation mark as not operator

The exclamation mark '!' (aka “Bang!”) is a negation operator, just
like the tilde '~':

```
     octave:1> if ! strcmp (program_name, "octave"),
     >   "It's an error"
     > else
     >   "It works!"
     > end
     ans = It works!
```

---

Next: Unwind-protect,
Previous: Exclamation mark as not operator,
Up: Octave Features

### 5.8 Increment and decrement operators

If you like the ++, += etc operators, rejoice!
Octave includes the C-like increment and decrement operators ++
and -- in both their prefix and postfix forms, in addition to
+=, -=, \*=, /=, ^=, .\*=,
./=, and .^=.

For example, to pre-increment the variable x, you would write
`++`x. This would add one to x and then return the new
value of x as the result of the expression. It is exactly the
same as the expression x `=` x `+ 1`.

To post-increment a variable x, you would write `x++`.
This adds one to the variable x, but returns the value that
x had prior to incrementing it. For example, if x is equal
to 2, the result of the expression `x++` is 2, and the new
value of x is 3.

For matrix and vector arguments, the increment and decrement operators
work on each element of the operand.

---

Next: Built-in ODE and DAE solvers,
Previous: Increment and decrement operators,
Up: Octave Features

### 5.9 Unwind-protect

Octave supports a limited form of exception handling modelled after the
unwind-protect form of Lisp. The general form of an
`unwind_protect` block looks like this:

```
     unwind_protect
       body
     unwind_protect_cleanup
       cleanup
     end_unwind_protect
```

Where body and cleanup are both optional and may contain any
Octave expressions or commands. The statements in cleanup are
guaranteed to be executed regardless of how control exits body.

The `unwind_protect` statement is often used to reliably restore
the values of global variables that need to be temporarily changed.

Matlab can be made to do something similar with their `OnCleanUp`
function that was introduced in 2008a.

---

Previous: Unwind-protect,
Up: Octave Features

### 5.10 Built-in ODE and DAE solvers

Octave includes LSODE and DASSL for solving systems of stiff ordinary
differential and differential-algebraic equations. These functions are
built in to the interpreter.

---

Next: Getting Octave,
Previous: Octave Features,
Up: Top

## 6 What documentation exists for Octave?

- Documentation- Getting additional help- User community- Bug reports

---

Next: Getting additional help,
Up: Learning more about Octave

### 6.1 What documentation exists for Octave?

The Octave distribution includes a 590+ page manual that is also
distributed under the terms of the GNU GPL.
It is available on the web at
http://www.octave.org/docs.html and you will also
find there instructions on how to order a paper version.

The complete text of the Octave manual is also available using the GNU
Info system via the GNU Emacs, info, or xinfo programs, or by using
the help -i command to start the GNU info browser directly from
the Octave prompt.

If you have problems using this documentation, or find that some topic
is not adequately explained, indexed, or cross-referenced, please send
a bug report to bug@octave.org.

---

Next: User community,
Previous: Documentation,
Up: Learning more about Octave

### 6.2 Getting additional help

If you can't find an answer to your question, the
help@octave.org mailing list is available for questions related
to using, installing, and porting Octave that are not adequately
answered by the Octave manual or by this document.

---

Next: Bug reports,
Previous: Getting additional help,
Up: Learning more about Octave

### 6.3 User community

To subscribe to the list, go to www.octave.org/archive.html and
follow the link to the subscription page for the list.

**Please do not** send requests to be added or removed from the
mailing list, or other administrative trivia to the list itself.

An archive of old postings to the help-octave mailing list is maintained
on http://www.octave.org/archive.html.

You will also find some user advice and code spread over the web. Good
starting points are the Octave Wiki http://wiki.octave.org and
Octave-Forge http://octave.sourceforge.net

---

Previous: User community,
Up: Learning more about Octave

### 6.4 I think I have found a bug in Octave.

“I think I have found a bug in Octave, but I'm not sure. How do I know,
and who should I tell?”

First, see the section on bugs and bug reports in the Octave manual.
When you report a bug, make sure to describe the type of computer you
are using, the version of the operating system it is running, and the
version of Octave that you are using. Also provide enough code so that
the Octave maintainers can duplicate your bug.

If you have Octave working at all, the easiest way to do this is to use
the Octave function `bug_report`. When you execute this function,
Octave will prompt you for a subject and then invoke the editor on a
file that already contains all the configuration information. When you
exit the editor, Octave will mail the bug report for you (in a unix-like
operating system).

If for some reason you cannot use Octave's `bug_report` function,
mail your bug report to bug@octave.org. Your message needs to
include enough information to allow the maintainers of Octave to fix the
bug. Please read the section on bugs and bug reports in the Octave
manual for a list of things that should be included in every bug report.

---

Next: Installation,
Previous: Learning more about Octave,
Up: Top

## 7 Getting Octave

- Source code- Pre-compiled binary packages- Octave for other platforms

---

Next: Pre-compiled binary packages,
Up: Getting Octave

### 7.1 Source code

Source code is available on the Octave development site, where you are
sure to get the latest version.

- http://www.octave.org/download.html- ftp://ftp.octave.org/pub/octave/

Since Octave is distrubted under the terms of the GPL, you can get
Octave from a friend who has a copy, by anonymous FTP, or by ordering
a tape or CD-ROM from the Free Software Foundation (FSF).

---

Next: Octave for other platforms,
Previous: Source code,
Up: Getting Octave

### 7.2 Pre-compiled binary packages

The Octave project does not distribute binary packages, but other
projects do. For an up-to-date listing of packagers, see:

- http://www.octave.org/download.html- http://wiki.octave.org/wiki.pl?CategoryInstall

As of today, Octave binaries are available at least on Debian, RedHat,
Suse and Fedora Linuxes, Mac OS X, Windows' 98, 2000 and XP.

---

Previous: Pre-compiled binary packages,
Up: Getting Octave

### 7.3 How do I get a copy of Octave for (some other platform)?

Octave currently runs on Unix-like systems, Mac OS X, and Windows.
It should be possible to make Octave work on other systems as well.
If you are interested in porting Octave to other systems, please contact
bug@octave.org.


---

Next: Common problems,
Previous: Getting Octave,
Up: Top

## 8 Installation Issues and Problems

Octave 3.2 require approximately 800MB of disk storage to unpack
and compile from source (considerably less if you don't compile with
debugging symbols). Once installed, Octave requires approximately 200MB
of disk space (again, considerably less if you don't compile with
debugging symbols).

- What else do I need?- Other C++ compilers?

---

Next: Other C++ compilers?,
Up: Installation

### 8.1 What else do I need?

To compile Octave, you will need a recent version of GNU Make. You
will also need GCC 3.3 or later, although GCC 4.1 or later is
recommended.

**You must have GNU Make to compile octave**. Octave's Makefiles
use features of GNU Make that are not present in other versions of make.
GNU Make is very portable and easy to install.

---

Previous: What else do I need?,
Up: Installation

### 8.2 Can I compile Octave with another C++ compiler?

Yes, but development is done primarily with GCC, so you may hit some
incompatibilities. Octave is intended to be portable to any standard
conforming compiler. If you have difficulties that you think are bugs,
please report them to the bug@octave.org mailing list, or ask
for help on the help@octave.org mailing list.

---

Next: How do I ...?,
Previous: Installation,
Up: Top

## 9 Common problems

This list is probably far too short. Feel free to suggest additional
questions (preferably with answers!)

- Octave takes a long time to find symbols.

  Octave uses the `genpath` function to recursively add directories
  to the list of directories searched for function files. Check the list
  of directories with the `path` command. If the path list is very
  long check your use of the `genpath` function.- When plotting Octave occasionally gives me errors like gnuplot> 9 0.735604
    line 26317: invalid command.

    There is a known bug in gnuplot 4.2 that can cause an off by one error
    while piping data to gnuplot. The relevant gnuplot bug report can be
    found at http://sourceforge.net/tracker/index.php?func=detail&aid=1716556&group\_id=2055&atid=102055

    If you have obtained your copy of Octave from a distribution please file
    a bug report requesting that the fix reported in the above bug report be
    included.- I cannot install a package. Octave complains about a missing `mkoctfile`.

      Most distributions split Octave into several packages. The script
      `mkoctfile` is then part of a separate package:
      - Debian/Ubuntu

        `aptitude install octave3.0-headers`- Fedora

          `yum install octave-devel`

---

Next: MATLAB compatibility,
Previous: Common problems,
Up: Top

## 10 How do I ...?

- How do I set the number of displayed decimals?

---

Up: How do I ...?

### 10.1 How do I set the number of displayed decimals?

```
     octave:1> format long
     octave:2> pi
     pi = 3.14159265358979
     octave:3> format short
     octave:4> pi
     pi = 3.1416
```

---

Next: Index,
Previous: How do I ...?,
Up: Top

## 11 Porting programs from Matlab to Octave

People often ask

“I wrote some code for Matlab, and I want to get it running under
Octave. Is there anything I should watch out for?”

or alternatively

“I wrote some code in Octave, and want to share it with Matlab
users. Is there anything I should watch out for?”

which is not quite the same thing. There are still a number of
differences between Octave and Matlab, however in general
differences between the two are considered as bugs. Octave might
consider that the bug is in Matlab and do nothing about it, but
generally functionality is almost identical. If you find a difference
between Octave behavior and Matlab, then you should send a
description of this difference (with code illustrating the difference,
if possible) to bug@octave.org.

Furthermore, Octave adds a few syntactical extensions to Matlab that
might cause some issues when exchanging files between Matlab and Octave
users. As both Octave and Matlab are under constant development the
information in this section is subject to change at anytime.

You should also look at the page
http://octave.sourceforge.net/packages.html and
http://octave.sourceforge.net/doc/ that has a function reference
that is up to date. You can use this function reference to see the
number of octave function that are available and their Matlab
compatibility.

The major differences between Octave 3.2.N and Matlab R2008a are:

- Nested Functions

  Octave doesn't yet have nested functions. That is

  ```
            function y = foo (x)
              y = bar(x)
              function y = bar (x)
                y = ...;
              end
            end
  ```

  There was discussion in Octave of having these even prior to Matlab,
  and the decision was made not to have these in Octave at the time for
  compatibility. The above written with sub-functions functions would be

  ```
            function y = foo (x)
               y = bar(x)
            end
            function y = bar (x)
               y = ...;
            end
  ```

  Now that Matlab has recently introduced nested functions, Octave will
  probably have them soon as well. Until then nested functions in Octave
  are treated as sub-functions with the same scoping rules as
  sub-functions.

  The authors of Octave consider the nested function scoping rules of
  Matlab to be more problems than they are worth as they introduce
  diffiult to find bugs as inadvertantly modifying a variable in a
  nested function that is also used in the parent is particularly easy.- Differences in core syntax
    There a few core Matlab syntaxes that are not accepted by Octave,
    these being
    - Some limitations on the use of function handles. The major difference is
      related to nested function scoping rules (as above) and their use with
      function handles.- Some limitations of variable argument lists on the LHS of an expression,
        though the most common types are accepted.- Matlab classdef object oriented programming is not yet supportted,
          though work is underway and when development more on to Octave 3.3 this
          will be included in teh development tree.
    - Differences in core functions
      A large number of the Matlab core functions (ie those that are in
      the core and not a toolbox) are implemented, and certainly all of the
      commonly used ones. There are a few functions that aren't implemented,
      for example `condest` or to do with specific missing Octave functionality
      (gui, dll, java, activex, dde, web, and serial functions). Some of the
      core functions have limitations that aren't in the Matlab
      version. For example the `sprandn` function can not force a
      particular condition number for the matrix like Matlab can.- Just-In-Time compiler
        Matlab includes a "Just-In-Time" compiler. This compiler allows the
        acceleration of for-loops in Matlab to almost native performance with
        certain restrictions. The JIT must know the return type of all functions
        called in the loops and so you can't include user functions in the loop
        of JIT optimized loops. Octave doesn't have a JIT and so to some might
        seem slower than Matlab. For this reason you must vectorize your code as
        much as possible. The MathWorks themselves have a good document
        discussing vectorization at
        http://www.mathworks.com/support/tech-notes/1100/1109.html.- Compiler
          On a related point, there is no Octave compiler, and so you can't
          convert your Octave code into a binary for additional speed or
          distribution. There is an example of how to do this at
          http://www.stud.tu-ilmenau.de/~rueckn/, but this is a very early
          example code and would need lots of work to complete it.- Graphic Handles
            Up to Octave 2.9.9 there was no support for graphic handles in Octave
            itself. In the 3.2.N versions of Octave the support for graphics
            handles is converging towards full compatibility. The `patch`
            function is currently limited to 2-D patches, due to an underlying
            limitation in gnuplot.- GUI
              There are no Matlab compatible GUI functions. There are a number of
              bindings from Octave to Tcl/Tk, Vtk and zenity included in the
              Octave Forge project (http://octave.sourceforge.net) for example
              that can be used for a GUI, but these are not Matlab
              compatible. Work on a matlab compatible GUI is in an alpha stage in the
              JHandles package (http://octave.sourceforge.net/jhandles/index.html).
              This might be an issue if you intend to exchange Octave code with
              Matlab users.- Simulink
                Octave itself includes no Simulink support. Typically the simulink
                models lag research and are less flexible, so shouldn't really be used
                in a research environment. However, some Matlab users that try to
                use Octave complain about this lack. There is a similar package to
                simulink for the Octave and R projects available at
                http://www.scicraft.org/- Mex-Files
                  Octave includes an API to the matlab MEX interface. However, as MEX is
                  an API to the internals of Matlab and the internals of Octave
                  differ from Matlab, there is necessarily a manipulation of the data
                  to convert from a MEX interface to the Octave equivalent. This is
                  notable for all complex matrices, where Matlab stores complex
                  arrays as real and imaginary parts, whereas Octave respects the C99/C++
                  standards of co-locating the real/imag parts in memory. Also due to the
                  way Matlab allows access to the arrays passed through a pointer,
                  the MEX interface might require copies of arrays (even non complex
                  ones).- Block comments
                    Block comments denoted by "%{" and "%}" markers are supported by
                    Octave with some limitations. The major limitation is that block
                    comments are not supported within [] or {}.- Mat-File format
                      There are some differences in the mat v5 file format accepted by
                      Octave. Matlab recently introduced the "-V7.3" save option which is
                      an HDF5 format which is particularly useful for 64-bit platforms where
                      the standard matlab format can not correctly save variables.. Octave
                      accepts HDF5 files, but is not yet compatible with the "-v7.3" versions
                      produced by Matlab.

                      Although Octave can load inline abd function handles saved by
                      Matlab, it can not yet save them.

                      Finally, Some multi-byte unicode characters aren't yet treated in
                      mat-files.- Profiler
                        Octave doesn't have a profiler. Though there is a patch for a flat
                        profiler, that might become a real profiler sometime in the future. see
                        the thread

                        http://www.cae.wisc.edu/pipermail/octave-maintainers/2007-January/001685.html

                        for more details- Toolboxes
                          Octave is a community project and so the toolboxes that exist are
                          donated by those interested in them through the Octave Forge website
                          (http://octave.sourceforge.net). These might be lacking in certain
                          functionality relative to the Matlab toolboxes, and might not
                          exactly duplicate the matlab functionality or interface.- Short-circuit & and | operators
                            The `&` and `|` operators in Matlab short-circuit when
                            included in an if statemant and not otherwise. In Octave only the
                            `&&` and `||` short circuit. Note that this means that

                            ```
                                        if (a | b)
                                          ...
                                        end
                            ```

                            and

                            ```
                                        t = a | b;
                                        if t
                                          ...
                                        end
                            ```

                            are different in Matlab. This is really a Matlab bug, but
                            there is too much code out there that relies on this behavior to change
                            it. Prefer the || and && operators in if statements if possible.

                            Note that the difference is also significant when either argument is a
                            function with side effects or if the first argument is a scalar and the
                            second argument is an empty matrix. For example, note the difference
                            between

                            ```
                                        t = 1 | [];          ## results in [], so...
                                        if (t) 1, end        ## in if ([]), this is false.
                            ```

                            and

                            ```
                                        if (1 | []) 1, end   ## short circuits so condition is true.
                            ```

                            Another case that is documented in the Matlab manuals is that

                            ```
                                        t = [1, 1] | [1, 2, 3];          ## error
                                        if ([1, 1] | [1, 2, 3]) 1, end   ## OK
                            ```

                            Also Matlab requires the operands of && and || to be scalar values but
                            Octave does not (it just applies the rule that for an operand to be
                            considered true, every element of the object must be nonzero or
                            logically true).

                            Finally, note the inconsistence of thinking of the condition of an if
                            statement as being equivalent to `all(X(:))` when X is a
                            matrix. This is true for all cases EXCEPT empty matrices:

                            ```
                                        if ([0, 1]) == if (all ([0, 1]))   ==>  i.e., condition is false.
                                        if ([1, 1]) == if (all ([1, 1]))   ==>  i.e., condition is true.
                            ```

                            However,

                            ```
                                        if ([]) != if (all ([]))
                            ```

                            because `samp ([]) == 1` (because, despite the name, it is really
                            returning true if none of the elements of the matrix are zero, and since
                            there are no elements, well, none of them are zero). But, somewhere
                            along the line, someone decided that if `([])` should be false.
                            Mathworks probably thought it just looks wrong to have `[]` be true
                            in this context even if you can use logical gymnastics to convince
                            yourself that "all" the elements of a matrix that doesn't actually have
                            any elements are nonzero. Octave however duplicates this behavior for if
                            statements containing empty matrices.- Solvers for singular, under- and over-determined matrices

                              Matlab's solvers as used by the operators mldivide (\) and mrdivide (/),
                              use a different approach than Octave's in the case of singular, under-,
                              or over-determined matrices. In the case of a singular matrix, Matlab
                              returns the result given by the LU decomposition, even though the underlying
                              solver has flagged the result as erroneous. Octave has made the choice
                              of falling back to a minimum norm solution of matrices that have been
                              flagged as singular which arguably is a better result for these cases.

                              In the case of under- or over-determined matrices, Octave continues to
                              use a minimum norm solution, whereas Matlab uses an approach that is
                              equivalent to

                              ```
                                        function x = mldivide (A, b)
                                          [Q, R, E] = qr(A);
                                          x = [A \ b, E(:, 1:m) * (R(:, 1:m) \ (Q' * b))]
                                        end
                              ```

                              While this approach is certainly faster and uses less memory than
                              Octave's minimum norm approach, this approach seems to be inferior in
                              other ways.

                              A numerical question arises: how big can the null space component become,
                              relative to the minimum-norm solution? Can it be nicely bounded, or can it be
                              arbitrarily big? Consider this example:

                              ```
                                        m = 10;
                                        n = 10000;
                                        A = ones(m, n) + 1e-6 * randn(m,n);
                                        b = ones(m, 1) + 1e-6 * randn(m,1);
                                        norm(A \ b)
                              ```

                              while Octave's minimum-norm values are around 3e-2, Matlab's results
                              are 50-times larger. For another issue, try this code:

                              ```
                                        m = 5;
                                        n = 100;
                                        j = floor(m * rand(1, n)) + 1;
                                        b = ones(m, 1);
                                        A = zeros(m, n);
                                        A(sub2ind(size(A),j,1:n)) = 1;
                                        x = A \ b;
                                        [dummy,p] = sort(rand(1,n));
                                        y = A(:,p)\b;
                                        norm(x(p)-y)
                              ```

                              It shows that unlike in Octave, mldivide in Matlab is not invariant
                              with respect to column permutations. If there are multiple columns of
                              the same norm, permuting columns of the matrix gets you different
                              result than permuting the solution vector. This will surprise many
                              users.

                              Since the mldivide (\) and mrdivide (/) operators are often part of a more
                              complex expression, where there is no room to react to warnings or flags, it
                              should prefer intelligence (robustness) to speed, and so the Octave developers
                              are firmly of the opinion that Octave's approach for singular, under- and
                              over-determined matrices is a better choice that Matlab's- Octave extensions
                                The extensions in Octave over Matlab syntax are
                                very useful, but might cause issues when sharing with Matlab users.
                                A list of the major extensions that should be avoided to be compatible
                                with Matlab are
                                - Comments in octave can be marked with #. This allows POSIX
                                  systems to have the first line as #! octave -q and mark the script
                                  itself executable. Matlab doesn't have this feature due to the
                                  absence of comments starting with #".- Code blocks like if, for, while, etc can be terminated with block
                                    specific terminations like "endif". Matlab doesn't have this and
                                    all blocks must be terminated with "end"- Octave has a lisp like unwind\_protect block that allows blocks of
                                      code that terminate in an error to ensure that the variables that
                                      are touched are restored. You can do something similar with
                                      `try`/`catch` combined with rethrow (lasterror ()) in
                                      Matlab, however rethrow and lasterror are only available in Octave 2.9.10 and later.

                                      Note that using `try`/`catch` combined with rethrow
                                      (lasterror ()) can not guarantee that global variables will be
                                      correctly reset, as it won't catch user interrupts with Ctrl-C. For
                                      example

                                      ```
                                                       global a
                                                       a = 1;
                                                       try
                                                         _a = a;
                                                         a = 2
                                                         while true
                                                         end
                                                       catch
                                                         fprintf ('caught interrupt\n');
                                                         a = _a;
                                                         rethrow (lasterror());
                                                       end
                                      ```

                                      compared to

                                      ```
                                                       global a
                                                       a = 1;
                                                       unwind_protect
                                                         _a = a;
                                                         a = 2
                                                         while true
                                                         end
                                                       unwind_protect_cleanup
                                                         fprintf ('caught interrupt\n');
                                                         a = _a;
                                                       end
                                      ```

                                      Typing Ctrl-C in the first case returns the user directly to the
                                      prompt, and the variable "a" is not reset to the saved value. In the
                                      second case the variable "a" is reset correctly. Therefore Matlab
                                      gives no save way of temporarily changing global variables.- Indexing can be applied to all objects in Octave and not just
                                        variable. Therefore `sin(x)(1:10);` for example is perfectly valid
                                        in Octave but not Matlab. To do the same in Matlab you must do
                                        `y = sin(x); y = y([1:10]);`- Octave has the operators "++", "–", "-=", "+=", "\*=", etc. As
                                          Matlab doesn't, if you are sharing code these should be avoided.- Character strings in Octave can be denoted with double or single
                                            quotes. There is a subtle difference between the two in that escaped
                                            characters like `\n` (newline), `\t` (tab), etc are
                                            interpreted in double quoted strings but not single quoted strings. This
                                            difference is important on Windows platforms where the "\" character is
                                            used in path names, and so single quoted strings should be used in
                                            paths. Matlab doesn't have double quoted strings and so they should
                                            be avoided if the code will be transfered to a Matlab user.

---

Previous: MATLAB compatibility,
Up: Top

## Appendix A Concept Index

- Additional help: Getting additional help
- Binaries: Pre-compiled binary packages
- Bug in Octave, newly found: Bug reports
- Compatibility with Matlab: MATLAB compatibility
- DASSL: Built-in ODE and DAE solvers
- Decrement operators: Increment and decrement operators
- DJGPP: Octave for other platforms
- EMX: Octave for other platforms
- FAQ for Octave, latest version: Top
- Flex: What else do I need?
- FSF [Free Software Foundation]: Why GNU Octave?
- GNU [GNU's not unix]: Why GNU Octave?
- GNU Bison: What else do I need?
- GNU g++: What else do I need?
- GNU gcc: What else do I need?
- GNU Make: What else do I need?
- How do I ... ?: How do I ...?
- Increment operators: Increment and decrement operators
- libg++: What else do I need?
- LSODE: Built-in ODE and DAE solvers
- Mailing lists, bug-octave: Bug reports
- Mailing lists, help-octave: Getting additional help
- Manual, for Octave: Bug reports
- Matlab compatibility: MATLAB compatibility
- MS-DOS support: Octave for other platforms
- Octave bug report: Bug reports
- Octave, building: Installation
- Octave, documentation: Documentation
- Operators, decrement: Increment and decrement operators
- Operators, increment: Increment and decrement operators
- OS/2 support: Octave for other platforms
- Pre-compiled binary packages: Pre-compiled binary packages
- Source code: Source code
- Tips and tricks: How do I ...?
- Unwind-protect: Unwind-protect
- VAX: Octave for other platforms
- VMS support: Octave for other platforms
- Windows support: Octave for other platforms

## Table of Contents

- Frequently asked questions about Octave (with answers)- Preface- 1 What is Octave?
      - 1.1 Who develops Octave?- 1.2 Why GNU Octave?- 1.3 What version should I use?- 1.4 On what platforms does Octave run?
      - 2 Licensing Issues
        - 2.1 If I write code using Octave do I have to release it under the GPL?- 2.2 Since the MEX interface allows plugins to be distributed under terms that are incompatible with the GPL, does this mean that you are encouraging people to to write non-free software for Octave?- 2.3 I wrote a program that links with Octave libraries and I don't want to release it under the terms of the GPL. Will you change the license of the Octave libraries for me?
        - 3 How can I cite Octave?- 4 What's new in version series 3.0.N and 3.1.N of Octave- 5 What features are unique to Octave?
              - 5.1 Functions defined on the command-line- 5.2 Comments with #- 5.3 Strings delimitted by double quotes "- 5.4 Line continuation by backslash- 5.5 Informative block closing- 5.6 Coherent syntax- 5.7 Exclamation mark as not operator- 5.8 Increment and decrement operators- 5.9 Unwind-protect- 5.10 Built-in ODE and DAE solvers
              - 6 What documentation exists for Octave?
                - 6.1 What documentation exists for Octave?- 6.2 Getting additional help- 6.3 User community- 6.4 I think I have found a bug in Octave.
                - 7 Getting Octave
                  - 7.1 Source code- 7.2 Pre-compiled binary packages- 7.3 How do I get a copy of Octave for (some other platform)?
                  - 8 Installation Issues and Problems
                    - 8.1 What else do I need?- 8.2 Can I compile Octave with another C++ compiler?
                    - 9 Common problems- 10 How do I ...?
                        - 10.1 How do I set the number of displayed decimals?
                        - 11 Porting programs from Matlab to Octave- Appendix A Concept Index

---


#### Footnotes

[1] Matlab is a registered trademark of The MathWorks,
Inc.

---
